# Morphological Characteristics and Correction of Long Tubular Bone Regeneration under Chronic Hyperglycemia Influence

**DOI:** 10.1155/2020/5472841

**Published:** 2020-04-06

**Authors:** Yevhenii S. Dudchenko, Olena S. Maksymova, Vasyl S. Pikaliuk, Dmytro V. Muravskyi, Ludmila I. Kyptenko, Gennadii F. Tkach

**Affiliations:** ^1^Sumy State University, Sumy, Ukraine; ^2^Lesya Ukrainka Eastern European National University, Lutsk, Ukraine

## Abstract

**Introduction:**

Unsatisfactory consequences of bone regeneration disorders in diabetes mellitus (DM) patients, their high prevalence, complication number, and difficulties in treatment require further study and deeper understanding of reparative osteogenesis mechanisms under chronic hyperglycemia and finding new effective and affordable approaches to their treatment. Therefore, the aim of our work was to study the histological, ultramicroscopic, and histomorphometric features of reparative osteogenesis in rats with chronic hyperglycemia (CH), as well as to investigate the possibility of platelet-rich plasma (PRP) use in a fracture area in order to correct the negative effects of CH on reparative osteogenesis processes. *Study Object and Methods*. The studies were performed on 70 white laboratory rats, mature males, which were divided into the following groups: control group, animals with posttraumatic tibial defect under conditions of CH exposure, rats with experimental CH that were administered with PRP into the bone defect, and animals for the assessment of glucose homeostasis and confirmation of simulated CH. Light microscopy was performed using an Olympus BH-2 microscope (Japan). Ultramicroscopic examination was performed using REM-102 scanning electron microscope. The statistical analysis was performed using SPSS-17 software package.

**Results:**

The formation of new bone tissue in animals with CH did not occur after two weeks. Only on the 30th day of reparative osteogenesis the newly formed woven bone tissue was 61.54% of the total regenerated area. It was less than the reference value by 22.89% (*P* < 0.001). On the 14th day of reparative osteogenesis, the regenerated area in a group of animals with CH and PRP injection consisted of connective tissue by 68.94% (4.94% less than in animals with CH (*P* < 0.001)) and woven bone tissue by 31.06%, (13.51% less than in the control group (*P* < 0.001)). On the 30th day, the area of woven bone tissue in a regenerate of this group was less than that of the control group by 12.41% (*P* < 0.001).

**Conclusion:**

Thus, chronic hyperglycemia contributes to inflammation delay within the bone defect site, which makes the process of reparative osteogenesis more prolonged. The results of chronic hyperglycemia effect on bone regeneration are also impairment of osteogenic cell proliferation and shift of their differentiation towards the fibrocartilage regenerate formation. The PRP corrects the negative impact of chronic hyperglycemia on reparative osteogenesis, promoting more rapid inflammatory infiltrate removal from the bone defect site and osteogenic beam formation and remodeling of woven bone into lamellar membranous bone tissue.

## 1. Introduction

According to world statistics, musculoskeletal injuries rank second place among disability and mortality causes [1]. According to the forecast of Lopes et al., the annual fracture number in Europe will increase by 28% by 2025 (from 3.5 to 4.5 million cases) [2]. Today, according to the WHO, around 422 million people worldwide have diabetes mellitus (DM). In the next 25 years, experts predict the rise of DM incidence to 629 million, which is a major socioeconomic problem [3, 4].

In total, the number of musculoskeletal injuries in healthy persons' clavicle fractures accounts for 17.5%; brachial bone, 8.2%; forearm bones, 60.8%; femur, 8.2%; and lower leg bones, 5.2% [5]. Among DM patients and persons with impaired glucose tolerance, forearm bone fractures account for 21%; vertebrae, 18%; femur, 18%; and lower leg bones, 19% [6]. Besides that, in DM patients, the proportion of delayed fragment consolidation, fissures, and false joints reaches from 8 to 32% compared to healthy individuals [7].

In recent years, autologous platelet concentrates have been widely used in clinical practice for tissue regeneration [8]. High efficiency of biological drugs based on platelet-rich plasma (PRP) in sports injury treatment and during operations on joints and bones in dentistry has been already proved [9, 10]. However, there is insufficient information about the efficacy and feasibility of PRP use in the treatment of long tubular bone fractures in persons suffering from chronic hyperglycemia or type 2 DM.

Therefore, unsatisfactory consequences of bone regeneration disorders in DM patients, their high prevalence, complication number, and difficulties in treatment require further study and deeper understanding of reparative osteogenesis mechanisms under chronic hyperglycemia and finding new effective and affordable approaches to their treatment.

The aim of this work was to study the histological, ultramicroscopic, and histomorphometric features of reparative osteogenesis in rats with chronic hyperglycemia, as well as to investigate the possibility of PRP use in a fracture area to correct the negative effects of chronic hyperglycemia on reparative osteogenesis processes.

## 2. Materials and Methods

70 white laboratory male rats (age—7-9 months) were used for experimental study. All animals were divided into the following groups: group I—control (animals with traumatic injury of the tibia) (20 rats); group II—animals with experimental CH and traumatic injury of the tibia (20 rats); group III—rats with experimental CH and traumatic injury of the tibia, which received PRP into the area of tibia fracture (20 rats); and group IV—animals for glucose homeostasis evaluation and confirmation of modeled CH (10 rats).

All animals were examined for their motor activity and condition of the outer covering. Then, rats were subjected to two-week quarantine. Experimental animals were in condition according to the general ethical principles of experiments on animals (Kyiv, 2001), Declaration of Helsinki (2000), and European Convention for the Protection of Vertebrate Animals used for Experimental and Other Scientific Purposes (Strasbourg, 1985). Ethics and morality were not violated during the research. Rats were in the vivarium room under constant temperature (24–25°C), humidity (60 ± 5) %, and 12-hour dark-light cycle. Current cell cleaning was performed daily.

CH in animals of groups II, III, and IV was modeled as follows. For 2 weeks, rats have been consuming 10% aqueous fructose solution instead of drinking water. After that, intraperitoneal administration of streptozotocin (Sigma-Aldrich, USA) (40 mg/kg) and nicotinic acid (1 mg/kg) was performed once for each rat. Control group animals were administered single intraperitoneal injection of sterile citrate buffer. Following the streptozotocin administration, animals were kept under normal vivarium conditions on normal diet for 60 days.

On the 60th day after CH modeling, fasting blood glucose, insulin, glycosylated hemoglobin and C-peptide were determined in animals of group IV. Acquired data was used to confirm the CH presence.

Holey defect of both tibias was modeled in group I, II, and III rats for further investigation of reparative osteogenesis at micro- and ultrastructural levels. Surgery was performed in aseptic conditions under ketamine (8 mg/kg) and xylazine (3 mg/kg) anesthesia. 30 minutes before surgery, animals were intramuscularly administered a prophylactic dose of ampicillin (7.5 mg/kg). Preoperative preparation of the surgical field was performed by shaving the wool in the area of the anterior surface of the tibia and three times treating with 3% alcohol iodine solution.

Soft tissue sections of 0.8-1.5 cm long were made along the margo anterior line of the tibia. Using a portable dental drill (sterile boron (*d* 1.6 mm), low revolutions with cooling) formed port into the bone marrow in the middle third of the tibial diaphysis. Surgical wounds were closed with skin suture treated with 3% alcohol iodine solution.

In group I and II animals, the bone defect was left to heal under the blood clot. In group III rats, in order to correct possible negative CH influence on reparative osteogenesis, PRP (dose—0.5 ml) was introduced into the wound before suturing. For this, previously, 2 ml of blood from the lateral tail vein was collected into 4 ml vacuum containers containing 0.35 ml of 10% sodium citrate solution. The lost blood volume was immediately restored by sterile saline infusion. The selected blood was centrifuged for 20 min at a speed of 2,000 rpm. As a result, two blood component fractions were observed in the test tube: the lower dark red fraction (cellular components) and the upper straw yellow fraction (serum components). After that, the contents of the upper fraction and upper portion of the lower fraction were pipetted and transferred to another tube. The resulting material was centrifuged for 15 min at a speed of 2,000 rpm, which led to formation of two fractions: the lower, platelet-rich plasma and the upper, platelet-poor plasma. The contents of the lower fraction were transferred to a sterile tube, and the volume was adjusted to 1 ml with 10% calcium chloride solution [11]. The resulting solution was injected into the wounds of animals.

Animals were removed from the experiment by the overdose of thiopental anesthesia (4 mg/100 g body weight) on the 14th and 30th day after trauma (these stages of bone healing process correspond to cell proliferation and differentiation, bone formation, and its adaptive restructuring).

To study the microscopic structure, the prepared portions of the left tibia with a defect were fixed in 10% formalin solution. Then, demineralization was carried out in 5% aqueous Trilon B solution. Further samples were dehydrated in alcohols of increasing concentration and poured into paraffin. Then, using microtome MC-2, sections from obtained preparations were made (thickness of 4-6 *μ*m). Staining was performed with hematoxylin-eosin. An Olympus BH-2 microscope (Japan) was used for light microscopy. The morphometric analysis was performed using a microgrid, microwave line, and Digimizer computing software (Version 5.3.5). The following parameters were measured: inflammatory infiltrate area (mm^2^), granulation tissue area (mm^2^), connective tissue area (mm^2^), bone tissue area (mm^2^), and cartilage area (mm^2^).

For ultramicroscopic examination using scanning electron microscopy, the injured right tibia of rats was removed and fixed in 2.5% glutaraldehyde solution (in 0.2 M cacodylate buffer with pH = 7.2 at +4°C) and postfixed in 1% OsO4 solution (for 4 hours at +4°C). Dehydration was done using series of ethyl alcohol ascending concentrations. Before examination, the specimens were sputtered with gold in vacuum post “VUP-5.” After that, specimens were placed in a scanning electron microscope “REM 102” and photographed.

Statistical processing of all obtained numerical data was performed using SPSS (version 17.0, Chicago, IL, USA). Validation for normality of distribution was implemented using the Kolmogorov-Smirnov criterion. Data are presented as mean (M) and standard deviation (SD). The significance of differences between two groups was determined using Student's criterion (*t*). The difference was considered significant if the probability of chance (*P*) did not exceed 0.05 (*P* < 0.05).

## 3. Results

The blood metabolic parameters in groups I (control) and IV (CH) are presented in Table 1. The fasting glucose (*P* < 0.001) and glycosylated hemoglobin (*P* < 0.001) were significantly higher in rats with CH compared to control animals. The insulin level was decreased in the CH group (*P* = 0.020), wherein the C-peptide amount was equal between two groups (*P* = 0.267). Thus, the obtained results confirm the presence of chronic hyperglycemia in experimental animals.

**Figure 1:**
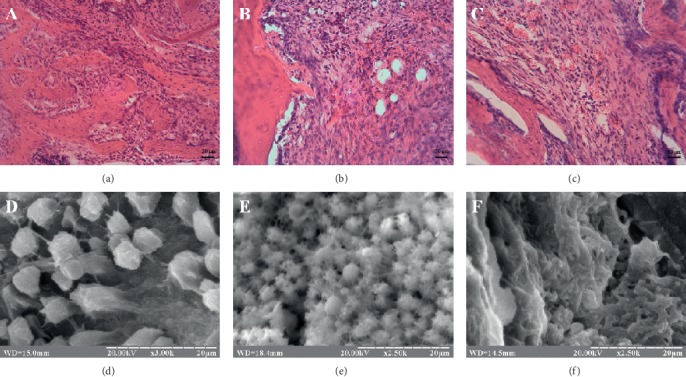
(a, d) Zone of tibial defect of control rats on the 14th day of reparative osteogenesis. (b, e) Zone of tibial defect of group II rats (animals with CH) on the 14th day of reparative osteogenesis. (c, f) Zone of tibial defect of group III rats (animals with CH and PRP injection) on the 14th day of reparative osteogenesis. (a–c) Hematoxylin and eosin stain. Magnification ×200. (d–f) Electronic scans.

The histological and ultramicroscopic analysis of reparative osteogenesis in the control group on the 14th day revealed bone beams with clearly separated young lacunae and fibroreticular tissue with high cell density of fibroblastic and osteoblastic diferons. The electronic scans of the osteogenic beam surface revealed a large number of spherical and cubic osteoblasts with long and thin processes. In most trabeculae, osteoblasts were immobilized in their own matrix (Figure 1(d)).

In animals with CH (group II), the defect area was filled with connective tissue. No complete wound cleaning from remnants of inflammatory infiltrate was observed. Local clusters of neutrophilic granulocytes, macrophages, lymphocytes, and adipocytes were localized in the central part of the defect (Figure 1(b)). In addition, scattered groups of chondrogenic cells were sometimes found among cellular infiltrates and collagen fibers. Electron scans of bone regeneration samples revealed clusters of rounded cells with numerous thin processes. Single deformed erythrocytes and thin fibers have been detected in the intervals between cell conglomerates (Figure 1(e)).

In group III, the ordering and transformation of connective tissue into osteoid beams were noted. Most intensively, this process took place near the maternal bone. The area of connective tissue with a large number of sinusoidal-type capillaries without signs of bone formation was observed in the defect center (Figure 1(c)). Electronic scans of the regenerate surface showed osteoblasts with thin long processes surrounded by amorphous matrix (Figure 1(f)).

**Figure 2:**
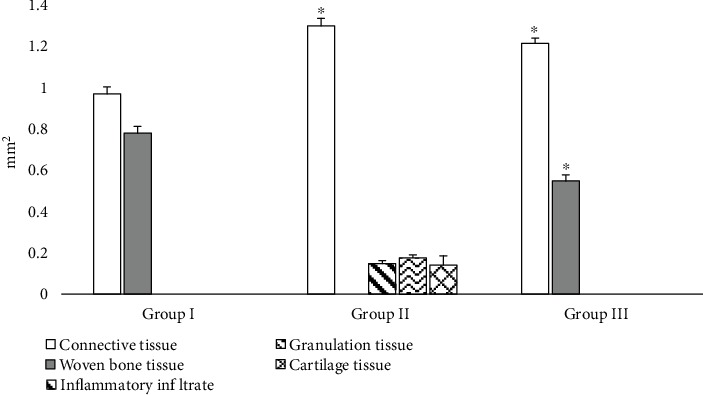
Areas of tissue-specific regenerate structures in bone defects of comparison groups on the 14th day of experiment: group I, control animals; group II, animals with CH; and group III, animals that were injected with PRP after tibial injury; ^∗^statistically significant differences between comparison groups (the control group was used as reference).

Figure 2 shows the tissue-specific composition of regenerate structures of bone defects on the 14th day of experiment. The posttraumatic area in the control group consisted of connective tissue (55.44%) and woven bone tissue (44.56%), wherein residual signs of inflammation were retained in CH rats. Inflammatory cell infiltration occupied 8.37% of the entire osteoreparation zone and granulation tissue area, 9.99%. In contrast to control animals, the woven bone tissue formation on the 14th day in CH animals did not occur. Instead, cartilage islets were found (7.96% of the total regenerated area). The rest of the regenerated area was connective tissue (73.68%). In a group of animals, which received PRP injection, the defect site was filled with connective tissue (68.94%) and small bone trabeculae (31.06%). No residual signs of inflammation, granulation, and cartilage were detected.

**Figure 3:**
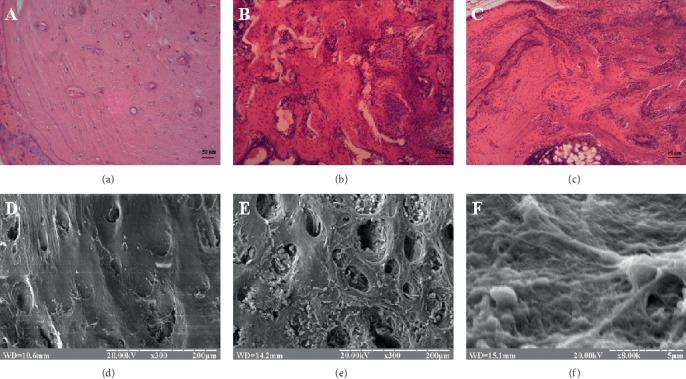
(a, d) Zone of tibial defect of control rats on the 30th day of reparative osteogenesis. (b, e) Zone of tibial defect of group II rats (animals with CH) on the 30th day of reparative osteogenesis. (c, f) Zone of tibial defect of group III rats (animals with CH and PRP injection) on the 30th day of reparative osteogenesis. (a–c) Hematoxylin and eosin stain. Magnification ×200. (d–f) Electronic scans.

On the 30th day of experiment, the bone defect in the control group was almost completely filled with newly formed bone tissue. At the same time, the processes of newly formed woven bone tissue remodeling into lamellar membranous bone tissue have begun. Active osteoclasts were observed around the bone beams. The new lamellar membranous bone tissue contained complete osteons with formed Haversian canals. Most intensively, these processes occurred near maternal bone. The central area of defect remained filled with woven bone tissue (Figure 3(a)). Electronic scanning of the defect area showed that its surface was covered with newly formed bone, penetrated by numerous transcortical vascular openings and osteoblastic lacunae (Figure 3(d)).

In group II, different sizes of osteogenic beams were formed. Wide gaps were observed between the trabeculae. Their formation near maternal bone was more structured. There were areas with not fully formed and interconnected beams. In the central part of defect, they had uneven thickness and disorganized placement. In addition, there was local replacement of defect by cartilage in the environment of woven bone tissue (Figure 3(b)). The formation of lamellar membranous bone tissue in this group did not occur. On electronic scans, the surface of the defect site had numerous intertrabecular gaps filled with cells and connective tissue. The fibrous structure of osteogenic beams was well visualized. Formation of complete periosteum of newly formed bone did not occur (Figure 3(e)).

In group III animals, more than half of the tibial defect was filled with woven bone tissue. Initial foci of lamellar membranous bone formation with restored osteons and Haversian canals were observed only near maternal bone (Figure 3(c)). Scanning microcopy revealed that the surface of defect central area was formed by massive collagen strands. Osteoblasts immersed in an osteoid matrix of bone beams were observed near maternal bone. The central zone of defect was filled with woven bone tissue and connective tissue. Regarding the periosteum, its formation on the 30th day of experiment in this group was just beginning (Figure 3(f)).

**Figure 4:**
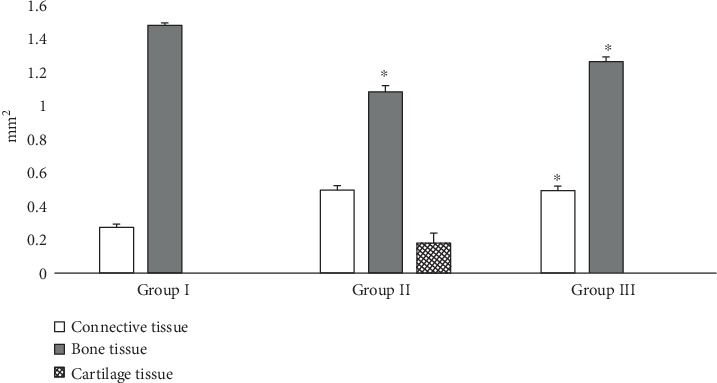
Areas of tissue-specific regenerate structures in bone defects of comparison groups on the 30th day of experiment: group I, control animals; group II, animals with CH; and group III, animals that were injected with PRP after tibial injury. ^a^Bone tissue—the summary area of different types of bone tissue (woven bone and lamellar membranous bone, for groups I and III; woven bone, for group II). ^∗^Statistically significant differences between comparison groups (the control group was used as reference).

Tissue-specific composition of bone regenerate structures on the 30th day of experiment is shown on Figure 4. In the control group, the defect area was filled with newly formed woven bone and lamellar membranous bone tissues (total area—84.44%). In rats with CH, the defect was filled with woven bone tissue (61.54%), connective tissue (28.22%), and cartilage tissue (10.24%). In group III, the formation of lamellar membranous bone had only just begun. The defect was mostly filled with woven bone (72.03%) and connective tissue (27.97%).

## 4. Discussion

In recent years, approaches to treatment of musculoskeletal injuries have changed significantly. According to ideas of most current authors, the use of PRP is simple and affordable and is a minimally invasive way to obtain natural concentration of autologous mediators, such as insulin-like growth factor-1, basic fibroblast growth factor, platelet-derived growth factor, epidermal growth factor, vascular endothelial growth, and transforming growth factor beta, which play a major role in inflammatory response attenuation and necrotizing cell elimination and have the number of potential advantages over existing methods. The availability, simplicity, efficiency of the method, and the absence of allergic reactions open the prospects for its further study and wider use in clinical medicine [8–10, 12, 13].

Research results report on high percentage (8 to 32%) of reparative osteogenesis disorders in type 2 DM patients and that hyperglycemia leads to decrease of proliferation and differentiation of cartilage and osteoblastic diferon cells involved in regeneration [14–17]. Our study revealed proliferation and differentiation disorders of osteoblastic diferon towards the formation of fibrocartilage regenerate in animals with CH.

Marin et al. and Hygum et al. showed that CH causes violation of coordinated action of signaling molecules and regulators of reparative osteogenesis, which leads to decreasing of osteoblast functioning, increasing of adipose tissue amount in regenerates, and significant inhibition of consolidation process [18, 19]. Our analysis of osteoreparation stages revealed delay in elimination of the first phase of inflammation in animals with CH. As a result, on the 14th day, the regenerate in this group contained fat cells and sections of local lymphocytic-leukocyte infiltration.

Insulin plays the important role in bone healing in DM patients through stimulation of bone matrix formation and increasing of collagen synthesis by osteoblasts. In vitro studies have identified decreasing of newly formed tissue ossification and impaired cartilage formation due to insulin deficiency. Researchers have found that collagen synthesis level in the fracture zone of DM rats decreased by 50-55%, which led to deterioration of mechanical properties of newly formed tissue [20]. In our study, we found impaired collagen structuring into osteoid beams and uneven formation and placement of cartilage in bone regenerates of animals with CH.

Dedukh and Sykal have reported about increasing of osteoclast density, increasing of the chondroid area, disorders of cartilage tissue replacement by bone, angiogenesis defects, and collagen and glycosaminoglycan synthesis impairment in the bone regenerate area of animals with DM [21]. Besides, the fibroreticular tissue area in bone regenerates of DM animals was significantly higher compared to that of control animals, which indicated the complication of reparative osteogenesis processes. Our morphometric analysis revealed that in animals with CH, the connective tissue area on the 14th day of reparative osteogenesis was higher by 18.24% (*P* < 0.001) and on the 30th day by 12.66% (*P* < 0.001), compared to the control group.

Animals with CH have longer reparative osteogenesis compared to animals with normal blood glucose. CH leads to neoangiogenesis impairment and inflammation augmentation, which finally cause disruption of osteogenic cell proper distribution and inhibit oxygen and nutrient entry to the regeneration zone. Also, tissue structure catabolism disorders and proliferation of adipogenic cells are observed in regenerated bone tissue of animals with DM. The abovementioned adipogenic cells increase the content of adipose tissue in the fracture region, which leads to inhibition of bone fragment consolidation [18, 22]. Our study confirms that reparative osteogenesis process is longer in animals with CH. On the 30th day of osteoreparation process, the area of newly formed woven bone tissue in animals with CH was 22.89% (*P* < 0.001) less compared to control.

In group III (animals with CH, which were injected PRP into the wound) on the 14th day of osteogenesis, residual signs of inflammation, granulation, and cartilage tissues in formed regenerates were not detected, unlike animals that were not administered PRP. On the 30th day, the area of woven bone tissue in regenerates of group III rats was larger compared to that of animals with CH, which were not administered PRP. Obtained data confirm that PRP treatment decreases damaged tissue swelling, inhibits acute inflammation, and promotes rapid shift from alteration phase to regenerative-reparative processes [23].

The abovementioned data also indicates antimicrobial activity of PRP, which is also shown in Bielecki et al. study [24]. Authors have analyzed the antibacterial effect of PRP in vitro. As a result, inhibition of Staphylococcus aureus and Escherichia coli growth and simultaneous induction of Pseudomonas aeruginosa growth were found. These data demonstrates different resistance of microorganisms to PRP. Authors believe that combination of inductive and antimicrobial PRP properties may improve the treatment outcomes of patients with infected fractures and false joints.

It should be said that there are few important limitations in our experiment, which did not allow us to evaluate the CH and PRP effect on bone regeneration more efficiently. Special staining methods with immunohistochemical reaction in order to recognize osteoblasts, endotheliocytes, and different types of leukocyte were not used. Thus, osteogenic cell amount, neoangiogenesis degree, and inflammation nature at the sites of bone regeneration were not accurately estimated. In addition, we have not applied PCR and immunohistochemistry methods for quantitative and qualitative assessment of molecular markers of bone regeneration (such as osteocalcin, bone sialoprotein, bone morphogenetic protein-2, and Runx2), which also did not allow us to describe the features of posttraumatic bone repair in different groups at a molecular level.

## 5. Conclusions

Thus, the chronic hyperglycemia contributes to inflammation delay within the bone defect site, which makes the process of reparative osteogenesis more prolonged. The results of chronic hyperglycemia effect on bone regeneration are also impairment of osteogenic cell proliferation and shift of their differentiation towards the fibrocartilage regenerate formation. The PRP corrects the negative impact of chronic hyperglycemia on reparative osteogenesis, promoting more rapid inflammatory infiltrate removal from bone defect site and osteogenic beam formation and remodeling of woven bone into lamellar membranous bone tissue.

## Figures and Tables

**Table 1:** The blood biochemical parameters in control and CH groups.

Parameter	Control (*n* = 10)	CH (*n* = 10)	*Р*
Fasting glucose (mmol/l)	4.89 ± 1.02	13.65 ± 2.21	<0.001
Insulin (*μ*MU/ml)	14.86 ± 2.51	12.67 ± 1.05	0.020
С-peptide (ng/ml)	3.51 ± 0.56	3.37 ± 0.45	0.579
HbA1c (%)	5.50 ± 1.01	7.36 ± 0.52	<0.001

Abbreviations: CH: chronic hyperglycemia; HbA1c: glycosylated hemoglobin. Data are presented as means ± SD.

## Data Availability

The data used to support findings of the research are available from the corresponding author upon request.
